# Insights into the nuclear-organelle DNA integration in *Cicuta virosa* (Apiaceae) provided by complete plastid and mitochondrial genomes

**DOI:** 10.1186/s12864-025-11230-8

**Published:** 2025-02-03

**Authors:** Seongjun Park, Yong Hwang, Heesoo Kim, KyoungSu Choi

**Affiliations:** 1 Institute of Natural Science, Yeungnam University, Gyeongsan, Gyeongbuk 38541 South Korea; 2 Biological Specimen Conservation Division, Diversity Conservation Research Department, Nakdonggang National Institute of Biological Resources, Sangju, Gyeongbuk 37242 South Korea; 3 Divesity Forecast & Evaluation Division, Diversity Conservation Research Department, Nakdonggang National Institute of Biological Resources, Sangju, Gyeongbuk 37242 South Korea; 4 Department of Biology, College of Natural Science, Kyungpook National University, Daegu, 41566 Korea

**Keywords:** Nuclear DNAs of plastid and mitochondrial origin, Intracellular DNA transfer

## Abstract

**Background:**

Gene transfer between the organelles and the nucleus plays a central role in shaping plant genome evolution. The identification and analysis of nuclear DNA of plastid (NUPTs) and mitochondrial (NUMTs) origins are important for exploring the extent of intracellular DNA transfer in genomes.

**Results:**

We report the complete plastid and mitochondrial genomes (plastome and mitogenome) of *Cicuta virosa* (Apiaceae) as well as a draft nuclear genome using high-fidelity (HiFi) PacBio sequencing technologies. The *C. virosa* plastome (154,449 bp) is highly conserved, with a quadripartite structure, whereas the mitogenome (406,112 bp) exhibits two chromosomes (352,718 bp and 53,394 bp). The mitochondrial-encoded genes (*rpl2*, *rps14*, *rps19*, and *sdh3*) were successfully transferred to the nuclear genome. Our findings revealed extensive DNA transfer from organelles to the nucleus, with 6,686 NUPTs and 6,237 NUMTs detected, covering nearly the entire plastome (99.93%) and a substantial portion of the mitogenome (77.04%). These transfers exhibit a range of sequence identities (80–100%), suggesting multiple transfer events over evolutionary timescales. Recent DNA transfer between organelles and the nucleus is more frequent in mitochondria than that in plastids.

**Conclusions:**

This study contributes to the understanding of ongoing genome evolution in *C. virosa* and underscores the significance of the organelle-nuclear genome interplay in plant species. Our findings provide valuable insights into the evolutionary processes that shape organelle genomes in Apiaceae, with implications for broader plant genome evolution.

**Supplementary Information:**

The online version contains supplementary material available at 10.1186/s12864-025-11230-8.

## Background

The transfer of genes from organelles to the nucleus is an important process continuing to influence eukaryotic genomic evolution [1]. Many genes originally present in the organelle genomes are transferred to the nuclear genome, resulting in highly reduced mitochondrial and plastid genomes [1]. Gene transfer has enabled the incorporation of critical organellar processes into the nuclear genome, resulting in more effective regulation, coordination, and protection from the mutagenic environments within the organelles [2–4]. Despite the extensive gene loss from organelles, previous studies suggested that organelle-to-nucleus gene transfer is an ongoing process in plant [1, 5]. Understanding intracellular DNA transfer (IDT) is critical because it can influence the genomic architecture, gene expression, and overall plant fitness. Despite their importance, the mechanisms and consequences of intracellular DNA transfer remain poorly understood and require further research.

In plant cell, plastid and mitochondrial genetic materials, including genes, can be transferred to nuclear genomes (nuclear DNA of plastid origin, NUPTs; nuclear DNA of mitochondrial origin, NUMTs) through IDT [1, 6]. Many of these transferred sequences may become non-functional; some retain or acquire new functions and are maintained by selection, leading to plastid- or mitochondrial-encoded gene loss in their genomes [7]. Functional gene transfer from organelles to the nucleus was observed in various plants [8], with the transferred genes playing critical roles in organelle biogenesis. Additionally, IDT also occurred between organelle genomes (plastid DNA of mitochondrial origin, PLMTs; mitochondrial DNA of plastid origin, MIPTs) [6]. PLMTs and MIPTs contribute to the complex evolutionary history of plant organelle genomes. MIPTs are widespread among angiosperms, whereas PLMTs are uncommon [6]. However, most IDT between organelles are non-functional [9].

*Cicuta virosa* L., commonly known as northern water hemlock, is a member of the family Apiaceae [10]. It is native to Northern and Central Europe, Northern Asia, and North America [11]. Its natural habitat is limited to a small area in Korea [12]. The *C. virosa* contains cicutoxin that disrupts the functioning of the central nervous system [13]. Apiaceae plastomes have a conserved quadripartite structure and gene content, although only the IR boundaries have shifted during the genome evolution [14–17]. Lineage-specific PLMTs were reported in five lineages of this family [18–22]. The Apiaceae represents an intriguing case for studying the dynamics of IDTs in angiosperms. However, a comprehensive understanding of the IDTs in this family requires a combination of organelle genomics. More than 100 Apiaceae species have complete plastome sequences (NCBI Genome Database, accessed November 4, 2024); however, little data exists on the mitogenome of this family.

Long reads generated by the ONT or PacBio platforms can increase the accuracy of nuclear or organelle genomic structures compared to those obtained by a short-read assembly [23–25]. To explore the extent and impact of intracellular DNA transfer events, we sequenced the complete organelle genomes and a draft nuclear genome of *C. virosa* using high-fidelity (HiFi) PacBio sequencing technologies. Using the draft *C. virosa* nuclear genome, we demonstrated successful functional gene transfer from the organelles to the nucleus. To improve our understanding of the mitogenomic evolution in Apiaceae, we compared the *C. virosa* mitogenome with the published mitogenomes from this family. The findings of this study advance our understanding of the ongoing processes of genome evolution in plants and provide a comprehensive view of the genomic interplay between organelles and the nucleus in *C. virosa*.

## Results

### Genome assembly

The genome of *Cicuta virosa* was sequenced using the PacBio platform, yielding 79 Gb of HiFi reads. A total of 2,558,827 reads were generated, with an average read length of 16,471 bp. Genome assembly using Hifiasm produced a draft of 1,265.91 Mb with an N50 contig size of 19.63 Mb across 2,746 contigs. High-coverage contigs revealed complete plastid and mitochondrial genomes with coverage depths of 24,030 × for the plastome and 309 × for the mitogenome. Of the raw HiFi reads, 15.04% and 2.62% were mapped into the plastome into the mitogenome, respectively.

### Organelle genome organization

**Fig. 1:**
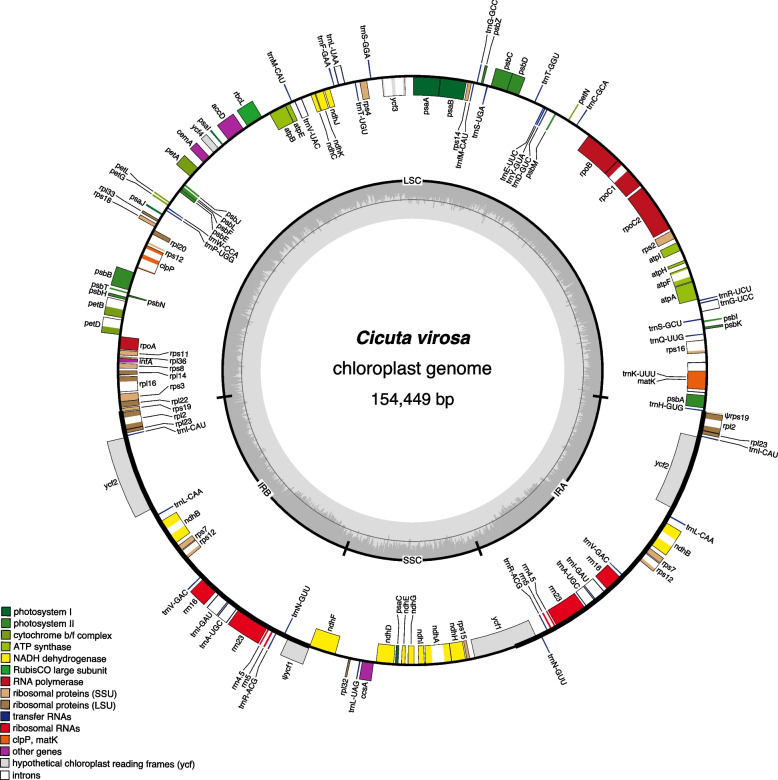
The *Cicuta virosa* plastome. Thick lines on the genome map indicate the inverted repeats, which separate the genome into small and large single-copy regions. Genes on the inside and outside of the map are transcribed in clockwise and counterclockwise directions, respectively

The *C*. *virosa* plastome was 154,449 bp in length, with a pair of inverted repeats (IRa and IRb) of 26,423 bp separated by small and large single-copy (SSC and LSC) regions of 17,545 and 84,059 bp, respectively (Table 1 and Fig. 1). The genome encodes 79 protein-coding genes, 30 tRNA, and four rRNA genes. The GC content of the plastome was 37.5%, and the genome contained five small repeat pairs (< 100 bp), accounting for 0.28% of the total sequence (Table S1). A comparison with the published *C. virosa* plastome (NC_037711, 154,569 bp) revealed a 99.6% sequence identity.

**Table 1:** General features of *Cicuta virosa* organelle genomes

	Plastome	Mitogenome	
Assembled contig(s)	1 circular	2 circulars	
Genome size (bp)	154,449	352,718	53,394
LSC (bp)	84,059	-	-
IR (bp)	26,423	-	-
SSC (bp)	17,545	-	-
GC content (%)	37.5	44.6	42
Protein genes	79	30	5
rRNA genes	4	3	0
tRNA genes	30	17	3
plastid-derived	-	23	1
Introns			
cis	20	18	2
trans	1	5	-
Repeat content (%)	0.28	28.48	8.97

**Fig. 2:**
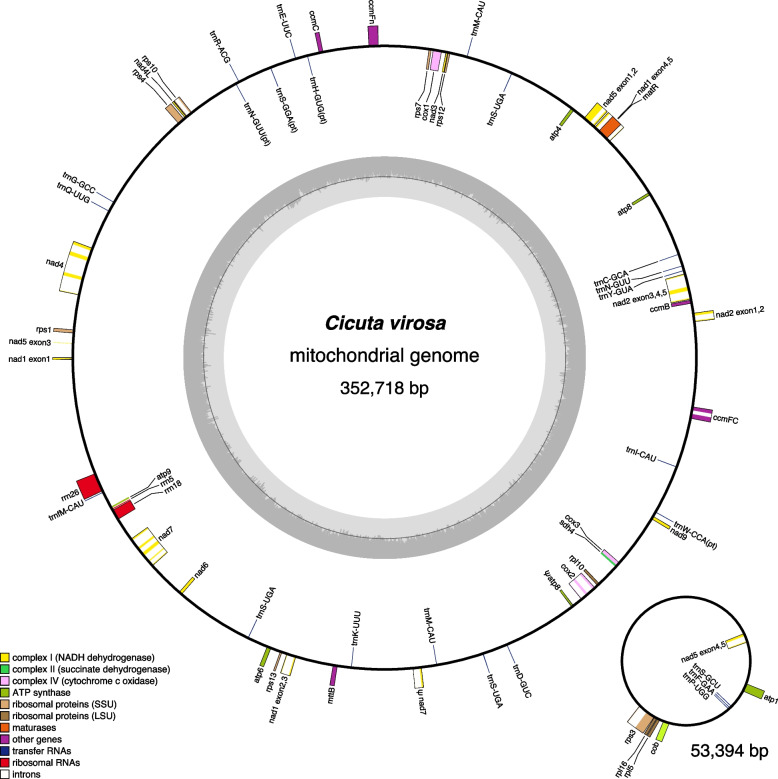
The *Cicuta virosa* mitogenome, represented as two circular maps (Chromosome 1 and Chromosome 2). Genes on the inside and outside of the map are transcribed in clockwise and counterclockwise directions, respectively

**Fig. 3:**
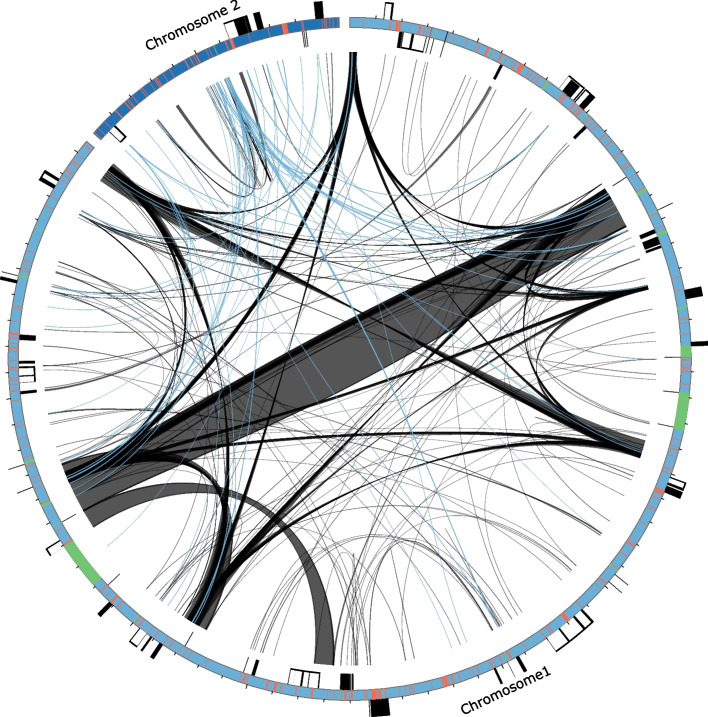
Distribution of repetitive DNA in the *Cicuta virosa* mitogenome. Black lines within circular maps indicate the positions of the pairs of repeats, with crossed connecting lines denoting reverse repeats. The blue lines within circular maps indicate the positions of the pairs of repeats between two chromosomes. Black boxes on the inner and outer circle indicate the positions of mitochondrial genes. Green and red lines inside of the circular maps indicate mitochondrial DNAs of plastid origin and transposable elements, respectively

The *C. virosa* mitogenome, totaling 406,112 bp, was assembled into two circular chromosomes of 352,718 bp and 53,394 bp (Table 1 and Fig. 2). It contains 58 genes, including 35 protein-coding genes, 20 tRNAs (five of which are plastid-derived), and three rRNAs. The mitogenome lacks the genes for the ribosomal proteins L2 (*rpl2*), S2 (*rps2*), S11 (*rps11*), S14 (*rps14*), and S19 (*rps19*), as well as succinate dehydrogenase 3 (*sdh3*). In the mitogenome, *cox1*, *nad4L*, and *rps4* begin with an ACG start codon instead of the standard ATG. PMGA predicted that RNA editing (C-to-U) modifies these start codons to the standard ATG. Additionally, RNA editing was predicted by PMGA to generate the stop codons in *atp6*, *atp9*, and *ccmFc*. The GC content was 44.6% in the larger chromosome and 42.0% in the smaller chromosome, with an overall GC content of 44.3%. The mitogenome contained 146 repeat pairs, accounting for 15.71% of the genome (Fig. 3 and Table S2). Of these, nine were large (> 1,000 bp) repeat pairs, ranging from 1,357 to 9,384 bp, with 17 intermediate (100–1000 bp) and 123 small (< 100 bp) repeat pairs in the mitogenomes. A total of 57 (ranging from 30 to 124 bp) spanned both chromosomes (Fig. 3; blue lines). The two chromosomes contained 19,163 bp (4.7%) and 3,603 bp (0.9%) of transposable elements (TEs), the majority of which were LTR retrotransposons (46.6% and 53.5%, respectively,) (Table S3). Twenty-one MIPTs were identified in the mitogenome, covering 5.60% of the genome, with fragments ranging from 57 to 9,193 bp (Table S4). These MIPTs contained eight intact protein-coding genes (*psbD*, *psbC*, *psaB*, *psbZ*, *psbL*, *rps14*, *rpl23*, *rpl2*), three intact rRNAs (23S, 4.5S, and 5S), nine intact tRNAs genes, several partial genes (*trnI*-CAU, *trnM*-CAU, *petB*, *petG*, *psaA*, *psbA*, *rps19*, *psbJ*, *psbF*, *rpoB*, and *ycf1*) and introns (*petD*, *ycf3*, and *trnI*-GAU), although only one fragment was present in the small chromosome. No PLMT hits were detected in reciprocal BLAST analysis.

### DNA transfers between organelle and the nucleus

Plastid- and mitochondrial-like sequences contigs were excluded from the initial nuclear genome assembly based on BLASTN analyses against the complete organelle genomes of *C. virosa*, resulting in 1,649 contigs totaling 1,219.2 Mb of the nuclear genome. The filtered nuclear genome was evaluated using BUSCO, which achieved 98.8% completeness based on eudicot genes, with only 0.3% fragmented genes and 0.9% missing genes.

To identify the NUPTs and NUMTs in the *C*. *virosa* nuclear genome, the plastome (containing one IR) and mitogenome (excluding MIPTs) were searched against draft nuclear contigs. For NUPTs, 6,686 hits were identified, covering 127.93 kb of the plastome (99.93%) and spanning 1,296.1 kb of the nuclear contigs (0.11%), with fragment lengths ranging from 50 to 15,195 bp (Table S5). The sequence identities of these NUPTs ranged from 80 to 100% compared to that in the plastid genome. The distribution of sequence identities among the NUPTs was as follows: 952 fragments showed identities greater than 99%, 3,305 fragments fell within the 95–100% range, 5,109 fragments ranged from 90–94%, 6,070 fragments were between 85–89%, and 6,686 fragments showed 80–84% identity. The average nucleotide sequence identity of the NUPTs was 93.64%. NUMTs returned 6,237 hits, covering 295.4 kb of the mitogenome (77.04%) and spanning 812.61 kb of the nuclear contigs (0.07%), with fragment lengths ranging from 50 to 5,114 bp (Table S6). The sequence identities of these NUMTs ranged from 80% to over 99% compared with those in the mitochondrial genome. The distribution of sequence identities for NUMTs was as follows: 1,351 fragments showed identities greater than 99%, 3,842 fragments were within the 95–100% range, 5,258 fragments were within the 90–94% range, 5,972 fragments were between 85–89%, and 6,237 fragments showed identities between 80–84%. The average nucleotide sequence identity of the NUMTs was 95.10%.

### Functional gene transfers between organelle and the nucleus

**Fig. 4:**
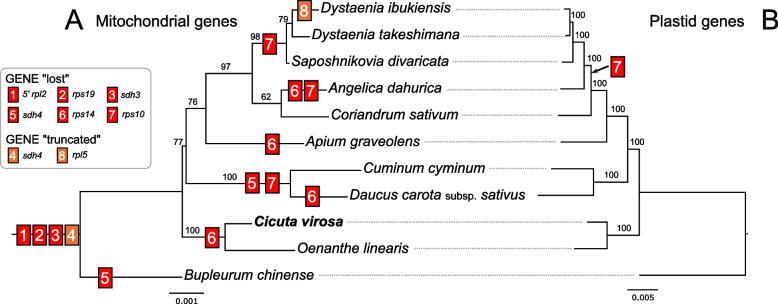
Phylogenetic distribution of gene content among 11 Apiaceae species, based on 23 mitochondrial genes (**A**) and 79 plastid genes (**B**)

A complete set of protein-coding genes was present in the *C. virosa* plastome; however, the losses of mitochondrial-encoded 5′ *rpl2*, *rps2*, *rps11*, *rps14*, *rps19*, and *sdh3* genes were identified in the mitogenome. Two ribosomal proteins, *rps2* and *rps11*, have been lost from the mitogenomes of nearly all core eudicots [8], and in this study, we focused on the loss of four other genes within the Apiaceae family. To investigate the evolutionary history of gene losses, we performed phylogenetic analyses based on 24 mitochondrial and 79 plastid genes (Fig. 4). The results indicated that three mitochondrial genes (5′ *rpl2*, *rps19*, and *sdh3*) were lost in a common ancestor of the analyzed Apiaceae species, while the loss of the *rps14* gene occurred independently across different lineages (Fig. 4). However, there was a discrepancy in the history of *rps10* gene loss. Specifically, based on the mitochondrial dataset, the loss of *rps10* occurred independently across the Apiaceae lineage. In contrast, the phylogram based on the plastid dataset placed *Coriandrum sativum* as strongly sister to the *Dystaenia/Saposhnikovia/Angelica* clade (BS = 100%), suggesting that the loss of *rps10* occurred in the common ancestor of the *Dystaenia/Saposhnikovia/Angelica/Coriandrum* clade (Fig. 4).

**Fig. 5:**
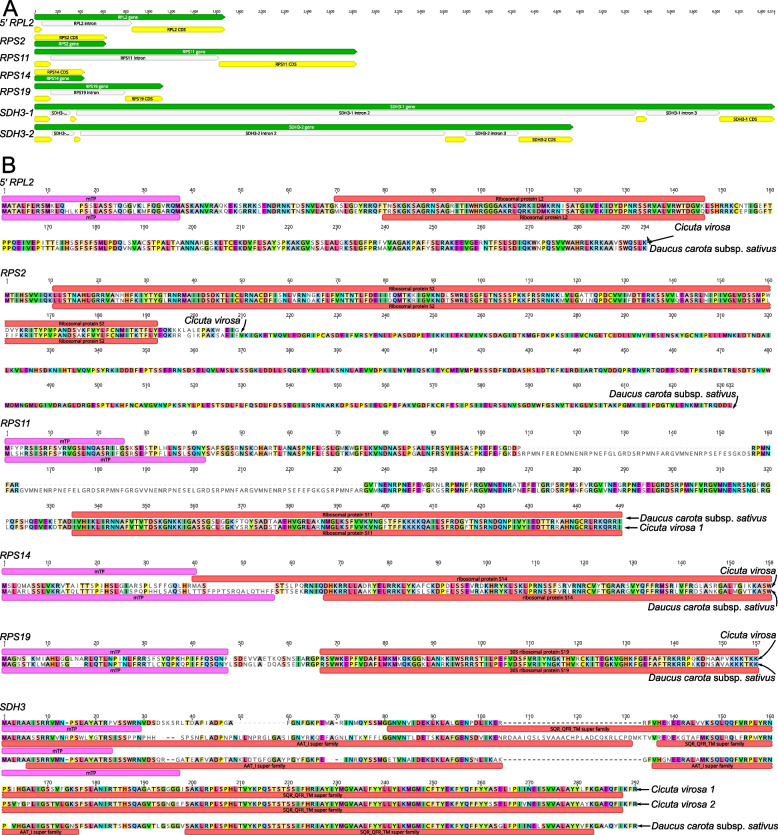
Mitochondrial gene transfer events to the *Cicuta virosa* nuclear genome. **A** Structure of five nuclear-encoded copies in *Cicuta virosa*. Genes, exons, and introns are represented by green, yellow, and white boxes, respectively. **B** Amino acid sequence alignments of nuclear-encoded organellar genes in *Cicuta virosa* and their nuclear copies in related species. Red boxes indicate the conserved domains, and pink boxes in the N-terminus indicate a transit peptide

To determine whether the four mitochondrial genes in *C. virosa* were transferred to the nucleus, the nuclear-encoded genes of *Daucus carota* subsp. *sativus* were queried against the draft *C. virosa *de novo nuclear genome sequences. We identified a nuclear gene for mitochondrial 5' *rpl2*, *rps2*, *rps11*, *rps14*, and *rps19* and two nuclear copies for mitochondrial *sdh3* (Fig. 5A and 5B). The nuclear-encoded genes ranged from 435 bp in *RPS14* to 6,514 bp in *SDH3-1* (Fig. 5A). The exon/intron patterns of the four nuclear-encoded genes were varied (Fig. 5A). For example, nuclear-encoded *RPS2* and *RPS14* has no introns, while 5′ *RPL2*, *RPS11*, and *RPS19* have two exons and *SDH3* has four.

A nuclear-encoded 5' *RPL2* gene was identified, showing nucleotide (nt) and amino acid (aa) sequence identities of 87.1% and 85.0%, respectively, to the *Daucus* 5' *RPL2* gene. The nuclear-encoded *RPS2* gene (627 nt and 208 aa) exhibited nt and aa identities of 83.0% and 85.2%. Similarly, the nuclear-encoded *RPS11* gene (2834 nt and 449 aa) showed nt and aa identities of 55.0% and 63.7%. For the nuclear-encoded *RPS14* gene (435 nt and 144 aa), the nt and aa identities were 67.7% and 62.0%. An open reading frame (ORF) for nuclear-encoded *RPS19* (468 nt and 155 aa) had nt and aa identities of 78.8% and 78.3%. Finally, two nuclear-encoded copies of *SDH3* (6,514 bp and 4,740 bp) exhibited aa identities of 78.2% and 61.0%, respectively. TargetP predictions indicated that all the genes contained mitochondrial targeting peptides (mTP), suggesting their possible mitochondrial localization (Fig. 5B). Specifically, mTPs were identified across each ORF of the four nuclear genes, with the first 23 to 46 amino acids at the N-terminal, and probabilities ranging from 0.2349 to 0.6729, with likelihoods from 0.6086 to 0.9911 (Table 2). However, TargetP did not identify evidence of mitochondrial targeting peptides in the nuclear-encoded *RPS2*. Interestingly, despite the absence of a transit peptide, the nuclear-encoded *RPS2* in *Arabidopsis* has been shown to be imported into the mitochondrion [26].

**Table 2:** Transit peptide prediction of nuclear-encoded ORFs

Gene	length (aa)	TargetP-2.0		
		Probability	likelihood	Tplen
*5' RPL2*	291	0.6146	0.8126	34
*RPS2*	208	-	0.2979	-
*RPS11*	449	0.2605	0.8334	42
*RPS14*	144	0.6729	0.6086	40
*RPS19*	155	0.5146	0.6144	36
*SDH3*	287	0.2349	0.9211	23
	250	0.3125	0.9911	28

### Comparison of *Cicuta* mitogenome to other Apicaceae

**Fig. 6:**
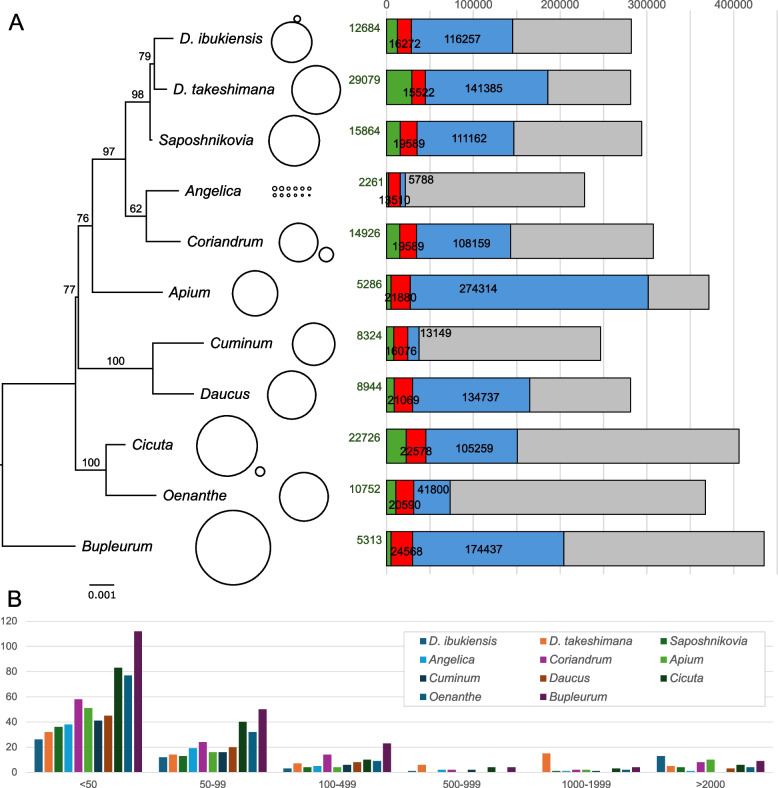
Genome size, amount of plastid-like and repetitive DNA and transposable elements in 11 Apiaceae mitogenomes. **A** Genome sizes, the number of bp of repetitive DNA (blue), plastid-derived sequences (green), and transposable elements (red). **B** Repeat size and frequency

The mitogenomes of the 11 Apiaceae species exhibited variable architectures and sizes (Fig. 6 and Table S7). For example, many mitogenomes have a single circular chromosome structure, however in *Angelica*, *Cicuta*, and *Coriandrum* mitogenomes, multi-chromosomal architectures consisting of two to 12 circular chromosomes were observed (Fig. 6). In *D. ibukiensis*, two circular chromosomes are connected by linear DNA molecules (Fig. 6), indicating a more complex organization of mitochondrial DNA. The mitogenome sizes of the 11 *Apiaceae* species ranged from 228,315 bp in *Angelica* to 435,023 bp in *Bupleurum* (Fig. 6 and Table S7). BLASTN searches of each mitogenome against its plastid counterpart revealed that MIPTs account for 0.99–10.33% of the 11 Apiaceae mitogenomes (Fig. 6 and Table S7). The 11 Apiaceae mitogenomes contain 5.52–7.49% TEs (Fig. 6 and Table S7). Repetitive DNA content among Apiaceae mitogenomes is highly variable, ranging from 2.54% in *Angelica* to 73.88% in *Apium* (Fig. 6 and Table S7). All Apiaceae had numerous small repetitive DNAs (< 100 bp), with the greatest number found in *Bupleurum* (Fig. 6 and Table S7).

To investigate the rates of mitochondrial DNA diversity in Apiaceae, we measured the amounts of shared DNA among the eight mitogenomes by performing BLASTN searches (e-value 1e^−10^) against *Cicuta*. This analysis revealed that *Cicuta* shared more sequences (214 kb) with *Oenanthe* than with other Apiaceae species, including *Bupleurum* (192.6 kb), *Daucus* (175.5 kb), *Cuminum* (185.4 kb), *Apium* (188.5 kb), *Coriandrum* (203.7 kb), *Angelica* (177.3 kb), *D. takeshimana* (190 kb), and *D. ibukiensis* (193.5 kb). The GC content across the 11 Apiaceae mitogenomes ranged from 44.3% in *Cicuta* and *D. ibukiensis* to 45.5% in *Cuminum*.

## Discussion

In this study, we generated a high-quality assembly of the *Cicuta virosa* plastome and mitogenome and the draft nuclear genome to elucidate the key features of its organelle structure, gene content, and DNA transfer dynamics. Our findings also provide insights into the organelle genome evolution within the Apiaceae, particularly in light of gene loss, DNA transfer to the nucleus, and structural diversity across related species.

The plastome of *C. virosa* exhibits a conserved structure and gene content compared to those of Apiaceae species [14–17]. The 99.6% sequence similarity with the previously published *C. virosa* plastome indicated a significant level of stability in this species. Minor variants may indicate ongoing evolutionary processes that facilitate adaptation to environmental stressors or result in subtle genetic alterations over time. Conversely, the *C. virosa* mitogenome displays significant dynamics in its structure and gene content. The Apiaceae mitogenomes exhibit considerable variability in their genomic architecture, ranging from a single circular chromosome to a multi-chromosomal configuration (Fig. 6). The *C. virosa* mitogenome exhibits two distinct mitochondrial conformations, similar to that of *C. sativum*. The gene losses for *rpl2*, *rps14*, *rps19*, and *sdh3* in the *C. virosa* mitogenome were in line with those documented in other Apiaceae species [20, 22, 27–33]. This suggests that gene loss in the mitogenomes frequently occurred within this family (Fig. 4). For example, the losses of *rpl2*, *rps19*, and *sdh3* occurred in the common ancestor of Apiaceae. Particularly, *rps14* appears to have been lost independently multiple times, reinforcing the idea that plant mitochondrial genomes constantly evolve, even among closely related species. The most parsimonious scenario for the *sdh4* events suggests that the gene was truncated in a common ancestor, followed by a stochastic loss of the s*dh4*.

One of the most interesting findings of this study is the extensive transfer of organelle DNA to the nuclear genome. In land plants, the total length of NUPTs varies from 50 kb (*Arabidopsis thaliana*) to 1,073 kb (*Oryza sativa* subsp. *japonica*), with an average size of 389.6 kb [34]. The total length of NUMTs ranges from 74 kb (*Physcomitrella patens*) to 834 kb (*Oryza sativa* subsp. *japonica*) [34]. The NUPTs of *C. virosa* (1,296.1 kb) is the highest value and NUMTs in *C. virosa* represent 812.6 kb, which is a very high level, although our nuclear genome was draft nuclear contigs. Our results are also consistent with the positive correlation between the larger genome and NUPT and NUMT contents [34, 35]. With more than 6,000 of NUPTs and NUMTs also detected in the *C. virosa* nuclear genome, covering almost the entire plastome (99.93%) and a significant portion of the mitogenome (77.04%) (Table S5 and S6), it is clear that DNA transfer from organelles to the nucleus is an important feature in plant genome evolution. These transferred sequences may play a role in nuclear genome expansion, possibly by providing raw materials for evolutionary innovation.

The variation in the nucleotide sequence identity between NUPTs and NUMTs (ranging from 80% to over 99%) in the *C. virosa* nuclear genome (Table S5 and S6) suggests multiple DNA transfer events from plastids or mitochondria to the nucleus at different evolutionary times [36]. The NUPTs and NUMTs with sequence identities greater than 99% are likely the result of recent transfers, because these sequences do not have sufficient time to accumulate mutations. Contrastingly, those with lower identity, especially in the 80–84% range, represent older transfers that have diverged more significantly due to the accumulation of mutations over time. Additionally, the higher number of recent NUMTs (1,351) suggests that mitochondria-to-nuclear transfers are more frequent than the recent plastid-to-nuclear transfers (952). Our findings suggest that DNA transfer between organelles and the nucleus is an ongoing process that occurs at different times and potentially influenced by factors such as mutation rates and selective pressures within the nuclear genome [1, 37, 38]. We also found many MIPTs (but no PLMTs), and this significant amount of plastid-to-mitochondria transfer suggests that the interactions between these two organelles continue to shape their genetic landscapes.

The discovery of nuclear-encoded genes for mitochondrial genes, such as *rpl2*, *rps2*, *rps11*, *rps14*, *rps19*, and *sdh3*, provides further evidence (Fig. 5). These nuclear-encoded genes have likely replaced their mitochondrial counterparts, suggesting that the loss of organellar genes is often compensated for by their transfer to the nucleus [1]. We observed variations in the exon–intron structures of these nuclear-encoded genes, suggesting that they have adapted to their new nuclear environment over time, maintaining essential mitochondrial functions through novel genetic regulation [7, 39].

## Conclusions

This study sequenced, assembled, and annotated the mitochondrial and plastid genomes of *C. virosa* as well as the draft nuclear genome, facilitating comparative organelle analyses within the Apiaceae family. The results of this study contribute to our understanding of the evolutionary dynamics of plant organelle genomes, particularly regarding organelle-to-nuclear DNA transfers. The high number of organelle-to-nuclear DNA transfers observed in this study suggests that these processes play a key role in shaping nuclear genomes over time, potentially contributing to functional innovation or genomic stability.

## Methods

### Genome sequencing, assembly and annotation

Fresh leaf tissue of *C. virosa* was collected from Hoengseon, Gangwon-do, Korea, and deposited in the herbarium of the Nakdonggang National Institute of Biological Resources (NNIBR) (specimen number: NNIBRVP 132277). Professor KyoungSu Choi (Department of Biology, College of Natural Science, Kyungpook University) identified the plant material. Total genomic DNA was extracted using a modified CTAB method [40]. The DNAs was used to construct the SMRTbell library. The library was sequenced using the PacBio Revio platform (Pacific Biosciences, CA, USA).

### Genome assembly, filtering, and annotation of the organelle genomes

The obtained HiFi reads were used to generate assemblies using Hifiasm v0.19.9-r616 [41].

Geneious Prime 2022.2 (www.geneious.com) used a BLAST-like method to identify plastid and mitochondrial contigs using *Liriodendron tulipifera* plastome and mitogenome sequences (NC_008326 and NC_021152) as queries. The detected organellar contigs were manually aligned, and a consensus genome sequence was established for each by tracking and checking them. The coverage depth of the entire plastome and mitogenome sequences was determined by mapping HiFi reads using BWA v0.7.17 [42]. To predict all the tRNA genes in the organelle genomes, we used ARAGORN v1.2.38 [43] and tRNAscan-SE v2.0.9 [44]. Circular or linear organellar genomes were generated using OGDRAW v1.3.1 [45].

The organelle genome sequences were deposited in GenBank with accession numbers, PQ421495 (plastome), PQ423759 (mitogenome 1), and PQ423760 (mitogenome 2).

### Comparative analyses

ROUSFinder.py [46] was used to identify repetitive sequences in *Cicuta* organelle genomes. Repeat pairs on genome maps were drawn with the Circos v0.69–8 [47]. To explore PLMTs and MIPTs, we did reciprocal "BLASTN" searches between the plastome and mitogenome with an e-value threshold of 1 × 10^–10^, with at least 80% sequence identity and a minimum length of 50 bp. RNA editing sites were predicted using PMGA [48] with default settings. The CENSOR online server [49] was used to scan the mitogenome for potential nuclear transposable elements (TEs) using the default parameters and "green plants" as a reference sequence source. To investigate NUPTs and NUMTs, nuclear-like contigs were first selected by excluding the plastid- and mitochondrial-like contigs using “BLASTN” searches between the plastome and mitogenome against the initial genome contigs, with at least 90% sequence identity and alignment coverage. The filtered nuclear genome contigs were examined for completeness of the assembly using Benchmarking Universal Single-Copy Orthologs (BUSCO) v5.7.1 [50] with the lineage “eudicots_odb10”. Then, we performed “BLASTN” searches between the plastome or mitogenome against the filtered nuclear genome contigs with an e-value cutoff of 1 × 10^–10^, requiring at least 80% sequence identity and a minimum length of 50 bp.

### Identification of intracellular gene transfer

To investigate potential intracellular gene transfer (IGT) to the nucleus, the amino acid sequences of nuclear-encoded 5′ *RPL2* (NC_030382; LOC108208767), RPS2 (NC_030389; LOC108202942), *RPS11* (NC_030381; LOC108225478), *RPS14* (NC_030384; LOC108216542), *RPS19* (NC_030384; LOC135152412), and *SDH3* (NC_030386; LOC108192842) from *Daucus carota* subsp. *sativus* genome (cultivar DH1 v3.0) were used to perform a “tBLASTn” (e-value cutoff of 1e^−10^) search against the filtered nuclear genome sequences of *Cicuta*. The conserved domain of the predicted open reading frame (ORF) was identified using the Conserved Domain Database (CDD) v3.19 [51]. TargetP v2.0 [52] were used to predict the presence of N-terminal sequences (chloroplast transit peptide [cTP] and mitochondrial targeting peptide [mTP]) and their potential cleavage sites.

### Phylogenetic analysis

To reconstruct the evolutionary relationships across the family Apiaceae, we used the protein-coding sequences of the 11 sequenced plastomes and mitogenomes (Table S8). Seventy-nine plastid and 24 mitochondrial protein-coding genes were shared among all 11 taxa sampled. Individual gene alignments were generated based on “Translation Align” option with MAFFT v. 7.490 [53] in Geneious Prime v2023.2.1 and each shared plastid and mitochondrial protein-coding genes were concatenated into a single alignment. Phylogenetic analyses were performed using IQ-TREE2 v2.2.03 [54] on the concatenated dataset (-m TEST and -B 1000).

## Supplementary Information

**Supplementary Material 1. **

## Data Availability

The organelle genome sequences were deposited in GenBank with accession numbers, PQ421495, PQ423759, and PQ423760.

## Abbreviations

NUPT: Nuclear DNA of plastid origin
NUMT: Nuclear DNA of mitochondrial origin
MIPT: Mitochondrial DNA of plastid origin
PLMT: Plastid DNA of mitochondrial origin
IGT: Intracellular gene transfer
IDT: Intracellular DNA transfer
